# Function of the Periosteum

**Published:** 1859-07

**Authors:** 


*Function of the Periosteum.*—M. Ollier has been recently experimenting on animals, to determine the functions of the periosteum.

He finds that when transplanted in the same animal it invariably produces bone, whether it is allowed to remain in connection with the uninjured tissue, or whether it is completely isolated. He wrapped it round muscles, implanted it under the skin of animals, in the comb of a cock, &c., and in every case bone was produced, the amount being greater the younger the animal. The texture of the bone resembles that of normal bone, containing medullary spaces which afterwards unite in one large cavity. The formative portion of the membrane consists of a layer of cells, or a blastema lying immediately below the fibrous portion of the periosteum. If this be scraped off, the germs of the future bone are destroyed, and no osseous tissue is formed.

